# Receptor for advanced glycation end products Glycine 82 Serine polymorphism and risk of cardiovascular events in rheumatoid arthritis

**DOI:** 10.1186/ar2175

**Published:** 2007-04-11

**Authors:** Lisa Carroll, Ian H Frazer, Malcolm Turner, Thomas H Marwick, Ranjeny Thomas

**Affiliations:** 1 Diamantina Institute for Cancer, Immunology and Metabolic Medicine, University of Queensland, Princess Alexandra Hospital, Ipswich Road, Brisbane, Queensland 4102, Australia; 2 Department of Medicine, University of Queensland, Princess Alexandra Hospital, Ipswich Road, Brisbane, Queensland 4102, Australia

## Abstract

Patients with rheumatoid arthritis (RA) are at risk of excess mortality, predominantly owing to cardiovascular (CV) events. The receptor for advanced glycation end products (RAGE) has been implicated in the perpetuation of the chronic inflammatory response in vascular disease. A Gly82→Ser polymorphism in the RAGE gene, which is associated with enhanced RAGE signaling, is present more frequently in patients with RA than the general population. To investigate whether RAGE Gly82→Ser polymorphism is associated with CV events in RA, we examined CV events, CV risk factors, features of RA and RAGE Gly82→Ser polymorphism in 232 patients with RA attending a tertiary referral hospital. CV events, the duration and severity of RA, and risk factors for CV disease were determined using patient questionnaires, chart review, laboratory analysis and radiographs. DNA was typed for HLA–DRB1 genes and RAGE Gly82→Ser polymorphism. The RAGE Ser82 allele, which is in linkage disequilibrium with the RA susceptibility allele HLA–DRB1*0401, was carried by 20% of patients. More than 20% of the cohort had suffered a vascular event; a shorter duration of RA, but not the RAGE genotype, was significantly associated with CV events. However, a history of statin use was protective. Thus, the RAGE Ser82 allele, associated with enhanced RAGE signaling, does not predispose to CV events in RA. However, treatment of hyperlipidemia with statins reduces the probability of a CV event.

## Introduction

The mortality of patients with rheumatoid arthritis (RA) is increased compared with the general population and most of this increase can be attributed to excess cardiovascular (CV) deaths [1]. Immune dysregulation and systemic inflammation have important roles in the accelerated atherosclerosis of RA. Serologic markers of inflammation, such as C-reactive protein (CRP) and serum amyloid A, are strongly associated with CV disease in the general population, in addition to systemic inflammatory diseases, including RA, systemic lupus erythematosus and inflammatory bowel disease [2-4]. Although the INTERHEART study showed 90% to 94% of the risk of myocardial infarction could be accounted for by conventional risk factors (abnormal lipids, smoking, hypertension, diabetes, abdominal obesity and inactivity), in addition to psychosocial factors and diet [5], the contribution of inflammation is associated with these risk factors. Inflammation might potentiate a prothrombotic inflammatory state, or promote metabolic and psychosocial factors, which affect disease [6,7]. For example, smoking is a risk factor for both CV disease and RA and itself associated with the promotion of inflammation, characterized by elevated CRP levels [8]. For patients with inflammatory polyarthritis or RA, the baseline level of CRP or peak erythrocyte sedimentation rate (ESR) within the first year after onset has been independently associated with the risk of CV death [9,10]. Thus, the progress of CV disease might depend on the burden of inflammation.

The receptor for advanced glycation end products (RAGE) is a multiligand member of the immunoglobulin superfamily of cell-surface receptor molecules encoded by chromosome 6 at the major histocompatability locus (MHC) class II/III junction [11]. When ligated, RAGE is implicated in atherogenesis and chronic inflammatory diseases by amplifying inflammatory events in tissues previously sensitized by lipid deposition or inflammatory triggers [12]. RAGE is expressed at low levels in normal tissues but upregulated at sites of vascular pathology or severe injury [13,14]. RAGE ligands include the calgranulin family of S100 proteins, the cytokine amphoterin (high-mobility group box chromosomal protein (HMGB1)), β-pleated sheet fibrils of amyloid and advanced glycation end products (AGEs) [15]. AGEs are the products of nonenzymatic glycation and oxidation of proteins that form with aging, diabetes and renal failure, and at sites of inflammation (by oxidation) [16].

Levels of S100 proteins and HMGB1 are increased in both adult and juvenile RA [17], and inhibition of the RAGE has been shown to suppress inflammation in animal models of arthritis [18]. There are four polymorphisms of the RAGE, of which the Gly82→Ser functional dimorphism shows the highest prevalence [19]. Ser82 enhances receptor signaling through mitogen activated protein (MAP) kinases and nuclear factor of kappa B (NF-κB) [18]. The increased prevalence of Ser82 in RA might be accounted for by linkage disequilibrium with the RA-associated HLA–DRB1*0401 allele [11,18,20]. However, although RAGE has been implicated in atherosclerosis, and the vascular component of diabetes mellitus and renal disease, RAGE Gly82→Ser polymorphism showed no association with CV disease in the general population [21]. Here, we tested whether the RAGE Gly82→Ser polymorphism was associated with CV events in RA.

## Materials and methods

### Patients

All current cases of RA, which met the American College of Rheumatology (ACR) 1987 revised criteria for the classification of RA [22], who presented for a scheduled appointment over a 5-month period (July to November 2003) at our tertiary hospital rheumatology clinic were invited to participate. Almost all RA patients regularly attending the rheumatology department, in addition to cases of recent onset, would be expected to have been captured during this timeframe. Patients completed a questionnaire detailing CV history, risk factors and their treatment, and details of RA and were clinically evaluated, with chart review for CV history, at least once. The study protocol was approved by the Princess Alexandra Hospital Research Ethics Committee (Brisbane, Australia).

### Genotyping

High-resolution HLA–DRB1 genotyping was carried out on buffy-coat DNA using PCR and sequence-specific oligonucleotide probes. To delineate the RAGE Gly82→Ser polymorphism, PCR was carried out as previously described [18]. Shared epitope was considered positive if at least one DRB1 allele was one of the RA susceptibility alleles described by Gregersen and co-workers [20].

### Assessment of cardiovascular events

To ascertain CV events, patients were asked for a history, dates and treatments of myocardial infarction (MI), angina, stroke, transient ischemic attack (TIA) or peripheral vascular disease (PVD), and these events were verified by reviewing the medical records. Although a number of patients had events before the diagnosis of RA, only CV events that occurred after RA diagnosis were included in the current analysis. Patients with multiple events had only one event counted per person. MI was identified if patients developed either of the following clinical signs: a typical rise and fall in the levels of biochemical markers consistent with myocardial necrosis, in addition to ischemic symptoms, development of pathologic Q waves on the electrocardiogram ECG and/or ECG changes indicative of ischemia (ST-segment elevation or depression); or either new pathologic Q waves on serial ECGs or pathologic changes of healed or healing MI [23]. Stroke or TIA was identified if patients had been admitted to the hospital with evidence of ischemic occlusion on computed tomography (CT) scanning, carotid endarterectomy, or symptoms of stroke/TIA, evidence of a significant plaque on carotid ultrasound and neurologic *sequelae*, with the exclusion of subarachnoid hemorrhage and space-occupying lesions. PVD was confirmed if Doppler ultrasonography showed significant large-vessel disease.

### Assessment of potential cardiovascular risk factors

Cigarette smoking was assessed by questionnaire, which included details about past and present smoking habits, the number of cigarettes smoked per day and the number of years the patient had been a smoker. Diabetes mellitus was classified as present if patients had been diagnosed by a physician or were taking antidiabetic medications at the time of the assessment. A family history of CV or cerebrovascular attack before the age of 65 years in first-degree relatives and those with an average prednisone dose greater than 10 mg/day were determined by questionnaire. If stroke was deemed hemorrhagic, the history was not included. The body-mass index (BMI) was calculated as weight in kilograms divided by the square of the height in meters. Blood pressure was measured at the time of evaluation. Hypercholesterolemia and hypertension were identified if the diagnoses were recorded in medical records by a physician or patients were taking lipid-lowering or antihypertensive drugs at the time of the evaluation. The percentage risk of CV events over the next 5 years was estimated using the 'CV disease risk calculator' derived from the Framingham study [24] for patients between 30 and 74 years of age and without a history of clinical CV disease.

Laboratory data collected at the time of clinical evaluation included the fasting total cholesterol level, low-density lipoprotein (LDL) level, high-density lipoprotein (HDL) level, very-low-density lipoprotein (VLDL) level, triglyceride (TG) level, LDL: HDL cholesterol ratio, glucose level, renal and liver functions, CRP level, ESR, anti-cyclic citrullinated peptide anti-CCP antibody and rheumatoid factor (RF) status. A 12-lead ECG carried out within the previous 12 months was scored for evidence of Q waves, to ascertain possible silent coronary disease. Creatinine clearance (CrCl) was estimated for each patient on the basis of the serum creatinine (SCr) level, age (in years) and ideal body weight (in kg), using the Cockcroft and Gault method, as follows:

CrCl (ml/min) = [(140 - age) × (ideal weight)]/833 × SCr (mmol/l). For females, the formula was multiplied by 0.85 [25].

### Assessment of rheumatoid arthritis radiographic severity

Hand radiographs carried out at the time of evaluation were scored for erosions and joint-space narrowing using the modified Sharp score [26].

### Statistical analysis

Analyses were conducted using standard software (Excel 2003, Microsoft, Redmond, WA, USA; and Graphpad Prism 4 and SPSS version 12, SPSS Inc., Chicago, IL, USA). Correlates of the RAGE Ser82 isoform were sought in a logistic regression model. Demographic factors, CV risk factors, RA features, the RAGE Ser82 isoform and HLA–DRB1 genotype were assessed univariately as predictors of the time from RA diagnosis to a CV event, using Cox proportional hazards regression to compute hazard ratios (HRs), with Bonferroni correction for multiple comparisons. All variables that were significant at *p *< 0.10 on univariate analysis were added to the multivariate model. Variables significant at *p *< 0.05 on backwards, stepwise regression analysis were retained.

## Results

### Cardiovascular events experienced by rheumatoid arthritis patients within this cohort

We studied 232 patients with RA who had a mean disease duration of 15 years and mean age of 61.8 years (range, 17 to 87 years) for CV events, duration and features of RA, CV disease risk factors, and HLA–DRB1 and RAGE Gly82→Ser genotypes. The characteristics of the patients are shown in Table 1. Of the patients studied, 94% were Caucasian. The sample included nine Asian patients, three Australian aboriginals and one patient of Pacific island origin. Previously defined CV risk factors were common. Of the patients studied, 63% were positive for RF and 76% had radiographic evidence of erosive disease (Table 1). Eighteen patients who had an onset of RA within the previous 12 months were included; RA had been present for between 1 and 64 years in the remaining patients. More than 20% of patients had experienced, or developed during the course of the study, a vascular thromboembolic syndrome, including MI, an episode of angina, stroke, TIA and diagnosis of PVD. MI and angina accounted for the majority of CV morbidity, although some patients had suffered both events (Table 1). Two patients died within 12 months of consent, both from vascular causes: an extensive ischemic stroke and a massive MI. The former patient died before blood collection for RAGE genotyping.

**Table 1:** Demographic details, CV risk factors, features of RA and RA disease control in the RA study population

**Demographics**	
Age (years)	61.8 (13.7)
Females, n (%)	147 (63.9)
Duration of RA (years)	15 (13)
RF positive, n (%)	141 (62.9)
**CV disease**	
History of MI, n (%)	24 (10.5)
History of angina, n (%)	21 (9.2)
History of stroke/TIA, n (%)	16 (7.0)
History of PVD, n (%)	10 (4.4)
Any vascular event, n (%)	47 (20.5)
**Risk factors for CV disease**	
Smoking pack-year history^a^	17 (22)
Current smoker, n (%)	52 (22.9)
History of hypertension, n (%)	78 (33.9)
History of hyperlipidemia, n (%)	52 (22.9)
Use of statins in hyperlipidemia	30 (14.8)
History of diabetes, n (%)	30 (13.3)
Family history CV disease, n (%)	58 (26.0)
**Clinical findings**	
BMI (kg/m^2^)	27.1 (6.4)
Systolic BP (mmHg)	133 (19)
Diastolic BP (mmHg)	78 (10)
**Laboratory tests**	
ESR (mm/h)	25 (22)
CRP (mg/l)	17.1 (27.4)
Total cholesterol (mmol/l)	5.2 (1.0)
HDL cholesterol (mmol/l)	1.5 (0.4)
LDL cholesterol (mmol/l)	3.0 (0.9)
LDL: HDL ratio	2.1 (0.8)
TG (mmol/l)	1.5 (1.0)
Homocysteine (mcmol/l)	12 (5)
Fasting glucose (mmol/l)	5.6 (1.6)
Serum creatinine (mmol/l)	0.08 (0.04)
CrCl (ml/min)	82.2 (31.2)
Framingham risk score (%)^b^	7.0 (5.9)
ECG evidence of ischemia,^c ^n (%)	7 (3.5)
**Severity and features of RA**	
Radiographic erosion score	22 (34)
Joint-space-narrowing score	21 (28)
Presence of erosive disease, n (%)	160 (76.0)
History of vasculitis, n (%)	20 (8.9)
Shared epitope,^d ^n (%)	144 (76.6)
>10 mg/day of prednisone, n (%)	17 (7.4)
RAGE polymorphism,^d ^n (%)	39 (20.4)

^a^Packets of cigarettes per day (20 cigarettes per pack) × no. of years of smoking (includes nonsmokers).

^b^Predicts the 5-year absolute risk of CV disease (MI, angina, coronary death, nonspecific heart disease, heart failure, PVD, stroke or TIA).

^c^4 out of 166 (2.4%) patients without known CV disease had ECGs suggestive of prior silent ischemia.

^d^191 patients were genotyped.

Except where indicated, values are the mean (± SD). BMI, body-mass index; BP, blood pressure; CRP, C-reactive protein; CV, cardiovascular; ECG, electrocardiogram; ESR, erythrocyte sedimentation rate; HDL, high-density lipoprotein; LDL, low-density lipoprotein; MI, myocardial infarction; PVD, peripheral vascular disease; RA, rheumatoid arthritis; RF, rheumatoid factor; RAGE, receptor for advanced glycation end products; SCr, serum creatinine; SD, standard deviation; TG, triglyceride; TIA, transient ischemic attack.

### Risk factors associated with cardiovascular events in rheumatoid arthritis

Cox regression was used to examine the association between CV events after onset of RA and CV risk factors, RA features and RAGE genotype (Table 2). Predictor variables were tested, considering the covariates as time-dependent during the period from onset of RA until the time of evaluation. A history of statin use for hyperlipidemia, a short disease duration and an average prednisone dose greater than 10 mg/day, but neither RAGE nor HLA genotype, were statistically significantly associated with CV events after RA diagnosis. Significant risk factors were further evaluated in multivariate Cox regression models (Table 3). All covariates were considered time-dependent. Significant associations persisted for disease duration and history of statin use but not prednisone dose.

**Table 2:** Univariate Cox regression analysis of CV risk factors and features of RA for risk of a time-dependent CV event among 232 patients

**Risk factor**	**Coefficient (β)**	**SE**	**Wald χ^2^**	**Risk ratio**	**95% CI**	**Corrected *p *value**^a^
Treatment with statins for hyperlipidemia	-1.06	0.317	11.192	0.35	0.19–0.65	<0.01
Average prednisone dose of >10 mg/day	2.71	0.65	17.2	15.03	4.18–54.08	<0.01
RA disease duration (risk per year)	-0.20	0.038	26.67	0.82	0.76–0.89	<0.01

^a^Bonferroni correction applied.

Differences were tested by univariate Cox analysis, with the dependent variable time to event (from diagnosis of RA). CI, confidence interval; CV, cardiovascular; RA, rheumatoid arthritis; SE, standard error.

**Table 3:** Multivariate Cox regression model for the risk of a time-dependent CV event among 232 patients

**Risk factor**	**Coefficient (β)**	SE	**Wald χ^2^**	**Risk ratio**	**95% CI**	**Corrected *p *value**
Statin therapy	-1.79	0.362	24.5	0.167	0.08–0.34	<0.0001
RA disease duration	-0.203	0.038	28.3	0.816	0.76–0.88	<0.0001

Backwards, stepwise Cox regression model, derived from significant univariate associations, including statin use, duration of arthritis and the dose of prednisone, the latter of which was not an independent predictor of outcome. CI, confidence interval; CV, cardiovascular; RA, rheumatoid arthritis.

### Receptor for advanced glycation end products Gly82→Ser polymorphism and rheumatoid arthritis

At least one RAGE gene with the Ser82 allele was present in 20% of RA patients in the present cohort. Only one patient was homozygous for the RAGE Ser82 isoform, thus it was not possible to evaluate a gene-dose effect. To further evaluate the association between the RAGE Ser82 allele and CV events, risk factors and RA characteristics, correlates of RAGE status were sought in a logistic regression model. The only correlate was the HLA–DRB1*0401 allele. Indeed, 87% of patients with the RAGE Ser82 isoform, but only 29% of patients with the RAGE Gly82 isoform, carried the HLA–DRB1*0401 allele on at least one chromosome (*p *< 0.0001). The patient homozygous for the RAGE Ser82 isoform was also homozygous for HLA–DRB1*0401. Carriage of any of the 'shared epitope' DRB1 alleles associated with RA susceptibility [20] was also associated with the RAGE Ser82 isoform, although less strongly. In combination, these data confirm that the RAGE Ser82 isoform and DRB1*0401 are in linkage disequilibrium in RA.

## Discussion

CV disease is a frequent and serious accompaniment for RA. We show here that in our population diagnosed with RA the risk of CV events was associated with history of statin therapy for hyperlipidemia and shorter RA disease duration, suggesting that the risk of vascular events is greatest in early disease. Association of CV events with shorter disease duration is consistent with several previous studies. Goodson and colleagues recently demonstrated 10% mortality, predominantly owing to CV events, within 10 years of onset of inflammatory polyarthritis [27]. In a large study of CV mortality in RA spanning 40 years of follow-up, disease duration was found to have no influence, suggesting that the increased risk of CV death was already present at RA diagnosis. Our data also demonstrate that the RAGE Ser82 allele, which is present in one-fifth of patients, does not contribute to the risk of CV events in patients with RA.

Laboratory, clinical and epidemiologic evidence indicates that immune dysregulation, in addition to ongoing systemic inflammation, is crucial to the excess atherogenesis and CV mortality in RA compared with age-matched individuals without RA [28-30]. Thus, baseline or average ESR or CRP levels over the course of RA have been shown to predict subsequent CV death [9,10]. However, inflammatory disease and its treatment, in turn, might affect either the prevalence or the contribution of traditional CV risk factors. Thus, cholesterol and inflammation tend to be inversely associated, owing to a disproportionate reduction of HDL and an adverse lipid profile [31,32]. Statins are known to be anti-inflammatory and have been shown to both improve vascular function in RA patients with a normal lipid profile and have additional immunomodulatory and RA disease-modifying benefits, including reduction of CRP levels, serum TNF concentrations and indices of plasma viscosity, in addition to short-term improvements in endothelial function and arterial stiffness [33-37]. Of clinical importance, this is the first study to show that statins significantly influence the outcome of atherosclerosis in RA. Although no direct indicator of inflammatory disease, such as CRP, persisted in the multivariate model, our study was limited in its ability to discern the influence of inflammation on CV events because we examined only RA patients and not matched non-RA controls and inflammatory markers and radiographs were examined at only a single time point, which occurred at variable times after commencing a variety of antirheumatic drugs rather than at baseline.

In RA, proinflammatory ligands of the RAGE, including S100A12 and HMGB1, are reported to be expressed at high levels, levels of soluble RAGE are reduced and RAGE expression is increased by synovial macrophages [17,38,39]. At least one RAGE allele was present as Ser82 in 10% to 20% of RA patients in the present prospective and retrospective cohorts, figures similar to previous studies of RA patients and higher than those in unselected population-based studies or control cohorts [18,19,21,40]. We confirm here that the RAGE Ser82 allele is in linkage disequilibrium with HLA–DRB1*0401 [40-42]. Because the RAGE Ser82 isoform is a functional polymorphism enhancing receptor signaling through MAP kinases and NF-κB, which activate an array of proinflammatory genes [18], we predicted an association of this polymorphism with CV events in RA, potentially through a more pronounced inflammatory response in these patients. However, the RAGE Ser82 isoform was not associated with more severe disease by any criteria in this RA cohort. Furthermore, no study has shown an independent association of the RAGE Ser82 isoform and RA. Thus, although certain RAGE ligands are highly expressed in RA and might contribute to the inflammatory process, the influence of the gain-of-function RAGE Ser82 isoform on RA expression is still unclear. Nevertheless, for similar reasons to those outlined above, the relationship between the RAGE Ser82 isoform and both inflammation and damage outcome in RA must be formally tested prospectively in RA inception cohorts.

Furthermore, in the current study of RA patients, the RAGE Ser82 isoform was not associated with CV events. This result supports the observation that the RAGE Ser82 isoform was not associated with CV disease in the general population [21], suggesting that the simple hypothesis that enhanced inflammatory signaling, mediated through the RAGE Ser82 isoform, predisposes to CV events is unlikely.

## Conclusion

A Ser82 polymorphic allele of RAGE associated with enhanced RAGE signaling is present in 20% of patients with RA in linkage disequilibrium with HLA–DRB1*0401. In a cohort of established RA patients attending a tertiary referral center, a history of statin use and shorter duration of RA, but not RAGE genotype, were significantly associated with CV events. Prospective trials to determine the role of statins on CV outcome in RA patients will be of great interest.

## Abbreviations

ACR = American College of Rheumatology; AGE = advanced glycation end product; BMI = body-mass index; CCP = cyclic citrullinated peptide; CrCl = creatinine clearance; CRP = C-reactive protein; CT = computed tomography; CV = cardiovascular; ECG = electrocardiogram; ESR = erythrocyte sedimentation rate; HDL = high-density lipoprotein; HMGB1 = high-mobility group box chromosomal protein; HR = hazard ratio; LDL = low-density lipoprotein; MAP = mitogen activated protein; MHC = major histocompatability; MI = myocardial infarction; NF-κB = nuclear factor of kappa B; PCR = polymerase chain reaction; PVD = peripheral vascular disease; RA = rheumatoid arthritis; RF = rheumatoid factor; RAGE = receptor for advanced glycation end products; SCr = serum creatinine; TG = triglyceride; TIA = transient ischemic attack; TNF = tumor necrosis factor; VLDL = very-low-density lipoprotein.

## Competing interests

The authors declare that they have no competing interests.

## Authors' contributions

LC, IF, MT, TM and RT were involved in conception, design, acquisition, analysis and interpretation of data. LC, IF, TM and RT drafted and revised the manuscript. All authors read and approved the final manuscript.
